# Promoter selectivity of the *Bacillus subtilis *response regulator DegU, a positive regulator of the *fla/che *operon and *sacB*

**DOI:** 10.1186/1471-2180-8-8

**Published:** 2008-01-15

**Authors:** Kensuke Tsukahara, Mitsuo Ogura

**Affiliations:** 1 Institute of Oceanic Research and Development, Tokai University, 3-20-1 Orido-Shimizu, Shizuoka 424-8610, Japan

## Abstract

**Background:**

The response regulator DegU and its cognate histidine kinase DegS constitute a two-component system in the Gram-positive soil bacterium *Bacillus subtilis*. Unphosphorylated and phosphorylated forms of DegU are known to activate target gene transcription in *B. subtilis*. Although phosphorylated DegU (DegU-P) regulates more than one hundred and twenty genes, the targets of unphosphorylated DegU are unknown, except for *comK*.

**Results:**

We found that the *fla/che *(flagella and chemotaxis) operon is positively regulated by unphosphorylated DegU. The effect was most prominent in a strain bearing the functional *swrAA *gene, a positive regulator of *fla/che*. Unphosphorylated DegU bound to two regions in the *fla/che *regulatory region containing an inverted repeat-like sequence that resembles the inverted repeat (IR) in the *comK *promoter. Mutational analysis revealed that positive regulation of *fla/che *by SwrAA requires DegU-binding. An analysis of the DegU-P-regulated gene *sacB *(levansucrase gene) by footprint and mutational analyses revealed that DegU-P bound to a direct repeat (DR) of the DegU-recognition motifs, which has been shown to be functional in vivo, while unphosphorylated DegU did not. These results strongly suggest that the arrangement of the DegU-binding motifs determines whether unphosphorylated DegU or DegU-P binds to the *sacB *promoter. The hypothesis was confirmed by observing *degS*-independent expression when the DR in the *sacB-lacZ *fusion was changed to an IR, suggesting that unphosphorylated DegU regulates the *sacB *promoter through the newly created IR. This was confirmed by binding of unphosphorylated DegU to the IR in the *sacB *promoter.

**Conclusion:**

This study demonstrated that DegU positively regulates *flgB *and *sacB *through its binding to the promoter regions. We demonstrated that DegU-P prefers binding to DR but not to IR in the *sacB *promoter.

## Background

To respond to environmental fluctuations, bacteria employ a large and elaborate family of two-component signaling systems. The classical two-component system consists of a sensor kinase and its cognate response regulator [1]. In response to the signal input, the kinase phosphorylates its own histidine residue. The phosphoryl group is then transferred to a conserved aspartate residue on the cognate response regulator, which then acts as a transcription factor in most cases. Given the many studies on how response regulator regulates output response by phosphorylation, it is not surprising that variable strategies were found [1]. Upon phosphorylation, some regulators dimerize to be activated or interact with other proteins or DNA [2], while other regulators are relieved from inhibition by their N-terminal domain [3]. One such two-component system in the Gram-positive soil bacterium *Bacillus subtilis *consists of the response regulator DegU and its cognate histidine kinase DegS. DegU belongs to the NarL family, whose members have a helix-turn-helix structure at their C-terminus [4]. The DegS-DegU system regulates many cellular processes, including exoprotease production and competence development [4-9]. It has also been reported to sense salt stress and to mediate appropriate responses [7,10,11]. In addition, it was found recently that the protein machinery for chromosome separation (SMC-ScpA-ScpB) forms a complex with DegS and inhibits its kinase activity [12]. The activity of DegU itself has been shown to be finely tuned by several factors. The Rap-Phr systems are regulatory machinery to receive extracellular signals [13]. DegU is negatively regulated by RapG since the protein inhibits its DNA-binding activity [14]. RapG activity is in turn inhibited by its cognate extracellular pentapeptide PhrG after the peptide is taken up by the cell. Thus, the RapG-PhrG system functions as a positive regulatory mechanism for DegU. Moreover, the transcription of *rapG *is repressed by RghR [15].

Unphosphorylated DegU is required for competence development and binds to the promoter region of *comK*, which encodes a master regulator of competence development [16,17]. Unphosphorylated DegU has also been reported to facilitate the binding of ComK to the *comK *promoter [18]. Previously we identified a DegU-recognized incomplete inverted repeat (IR) on the *comK *promoter (GTCATTTA-N7-TAAATATC) by using various mutated *comK-lacZ *fusions [19]. Additional targets of unphosphorylated DegU have not been identified.

Phosphorylated DegU (DegU-P) activates the expression of more than one hundred twenty genes, including *aprE *(which encodes alkaline protease) and *sacB *(which encodes levansucrase); it also represses *wapA*, which encodes a cell-wall associated protein [6-9,20]. In addition, the expression of *bpr*, which encodes bacillopeptidase F, has been reported to be probably dependent on DegU-P [8,21]. To date, the DNA recognition sequence of DegU-P has not been identified with the exception of *aprE *and *bpr*. Our analysis revealed that an important *cis*-factor for DegU-dependent *aprE *expression is a direct repeat (DR) of the downstream half of the DegU-recognized IR in the *comK *promoter with two-nucleotide spacing (-70 to -52 relative to the transcription start site), [19]. In addition, we identified three DRs with zero or two-nucleotide spacing, which are important for DegU-binding to the *bpr *promoter region and DegU-P-dependent expression of *bpr *[22]. Furthermore, overproduction of DegU or the *degU32 *mutation, which renders DegU-P resistant to dephoshorylation, resulted in a decrease in the expression of the *fla/che *operon encoding chemotaxis-related proteins and components of the flagella apparatus [5,8,9,23,24].

Since DegU-P stimulates the transcription of many genes and unphosphorylated DegU is required for *comK *transcription, DegU is regarded as a molecular switch that controls cell fate [5]. However, what factor determines promoter selectivity of DegU-P and unphosphorylated DegU remains unclear.

In this paper, we found that *flgB*, which is the first gene of the 26-kb-long *fla/che *operon, is subject to direct positive regulation by unphosphorylated DegU through two DegU-binding sequences containing an IR-like sequence. In contrast, footprint analyses of the *sacB *promoter revealed that DegU-P bound to a DR within the DegU-binding sequence. Since unphosphorylated DegU bound to *flgB*, the arrangement of the DegU-binding sequence within promoter regions (DR or IR-like sequence) must dictate which form of DegU (phosphorylated or unphosphorylated) binds to them. This hypothesis was confirmed by expression analysis of *sacB-lacZ *fusions carrying an artificial IR within their DegU-binding sequence.

## Results

### Unphosphorylated DegU binds to two IR-like sequences in *flgB*

**Figure 1:**
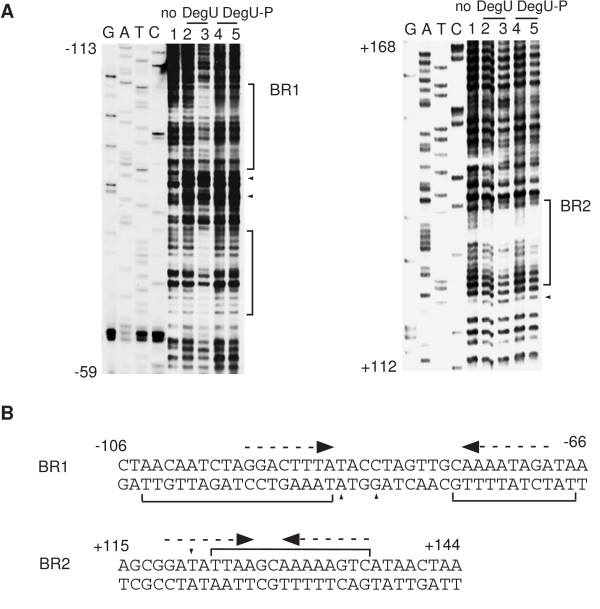
Footprint analysis of the *flgB *promoter. A. The probes were prepared by PCR amplification. To analyze the upstream (-120 to +38, the bottom strand in the left panel) and downstream (+15 to +264, the top strand in the right panel) regions, the oligonucleotide pairs flgB-F and flgB-D-bio, and flgB-DU-bio and flgB-DD, respectively, were used. The sequencing ladder templates for the upstream and downstream region analysis were generated by PCR using the oligonucleotide pairs flgB-F and flgB-D, and flgB-DU and flgB-DD, respectively. The probes (40 nM) were incubated with increasing amounts of DegU and His-DegS in the presence or absence of ATP, and subjected to DNase I cleavage. The sequencing ladder is shown in lanes G, A, T and C. The brackets and arrowheads indicate the protected regions and hypersensitive sites, respectively. 1. No protein. 2 and 4, 0.15 μM of DegU and 0.05 μM His-tagged DegS; 3 and 5, 0.3 μM of DegU and 0.1 μM His-tagged DegS. 2 and 3, no ATP; 4 and 5, 1 mM ATP. B. The nucleotide sequences of the regulatory regions are shown. The numbers indicate the nucleotide positions relative to the transcription start site. Dotted arrows above the sequences indicate the putative DegU-binding motifs.

**Figure 2:**
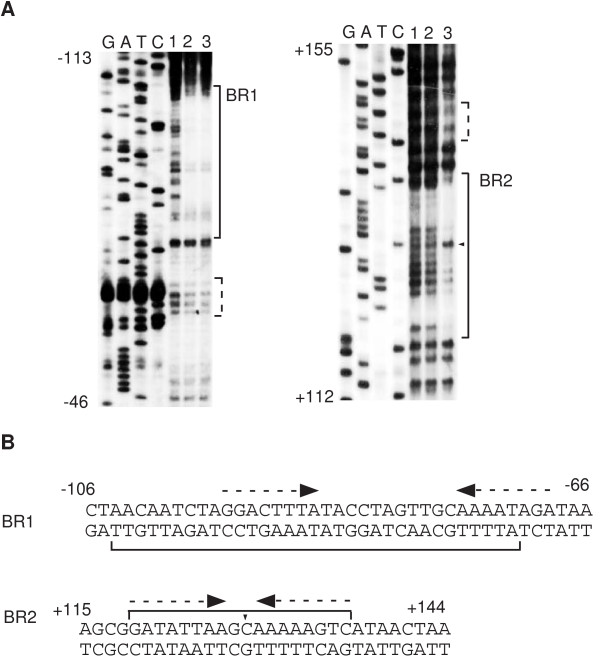
Footprint analysis of the *flgB *promoter using His-DegU. A. Preparation of the probes and sequencing ladders was the same as that in Figure 1. For footprint assays, we used the procedures developed for biotinylated DNA probes as described previously [47]. The sequencing ladder is shown in lanes G, A, T and C. The brackets, dotted brackets and arrowheads indicate the protected regions, weakly protected regions and hypersensitive sites, respectively. 1. No protein. 2, 0.15 μM of His-DegU; 3, 0.3 μM of His-DegU. No His-tagged DegS was added. B. The nucleotide sequences of the promoter region are shown. Dotted arrows above the sequences indicate the putative DegU-binding motifs. The numbers indicate the nucleotide positions relative to the transcription start site.

While studying the interaction of DegU with its target promoters, we found that unphosphorylated DegU bound to two DegU-binding regions present within *flgB *(BR1 and BR2). BR1 seems to contain an IR-like sequence (the -95 to -69 region relative to the transcription start site) with long spacing (eleven bases), and BR2 also seems to contain an IR-like sequence (the +119 to +136 region relative to the transcription start site) with two-nucleotide spacing (Figure 1). Next, we performed footprint analysis using His-tagged DegU and obtained essentially similar protection profiles to those obtained by using untagged DegU (Figure 2). Previously, gel retardation analysis using unphosphorylated His-tagged DegU showed that DegU bound to an upstream region from the transcription start site of *flgB*, although the binding sequence was not identified [24]. It should be noted that each of the half sites of these two IR-like sequences shows similarity to the DegU-recognition sequences in the *comK *promoter, although they are not perfect IR sequences. Furthermore, phosphorylation of DegU by His-tagged DegS abrogated its binding to BR1 but not that to BR2. Based on these results, we concluded that unphosphorylated DegU binds to two regions containing the IR-like sequence in the *flgB *regulatory region.

### DegU and SwrAA regulation of the expression of a F1D fusion carrying two BRs

**Figure 3:**
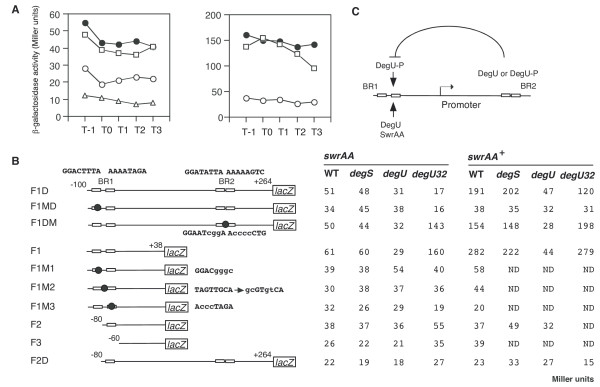
DegU-regulation of *flgB*. Cells were grown in MC medium. Their β-galactosidase activities were determined as described previously [45]. A. Left (*swrAA*) and right (*swrAA*^+^) panels show representative results of the β-galactosidase assays. All strains carry the *flgB-lacZ *(F1D) fusion. Filled circles, wild type (OAM385 in the left and OAM386 in the right); squares, *degS *(OAM388 in the left and OAM390 in the right); open circles, *degU *(OAM387 in the left and OAM389 in the right); triangles, *degU32*, (OAM391 in the left). The numbers on the x axis represent the growth time in hours relative to the end of vegetative growth (T0). B. Transcription of various *flgB-lacZ *fusions in each genetic background. The open rectangles and filled circles on the line indicate the DegU-binding motifs and introduced mutations, respectively. The mutant fusions tested included the F1 to F3 series of fusions (the 3' ends are +38) as well as F1D and F2D (the 3' ends are +264), in which increasing deletions were made from the 5' end, and the M series, in which point mutations (small letters indicate replaced nucleotides) were introduced. F1MD and F1M1 contain the same mutation. All fusions were constructed by cloning various PCR products into pIS284. Consequently, all strains are derivatives of OAM385 (*amyE*::*flgB-lacZ*). The oligonucleotides used to construct the pIS284 derivatives are shown in Additional file 1. The activities were assayed hourly from T-1 through to T3. At least three independent assays were performed and the averages of the peak values are shown. Standard deviations did not exceed 20%. The "*degS*", "*degU*" and "*degU32*" columns show the β-galactosidase activities of each fusion in the *degS*::Em^r^, *degU*::Km^r ^and *degU32 *backgrounds, respectively. ND means no data. C. Schematic representation of the regulation of *flgB*. Arrows and a curved T-bar indicate activation and inhibition, respectively.

To examine the in vivo functions of the BRs, we constructed a F1D fusion carrying both BRs and measured the β-galactosidase activities of the fusion in various genetic backgrounds, including disruption mutation of *degS *or *degU *in the presence of active or inactive *swrAA *(Figure 3). The *degS *mutation is an in-frame deletion, which results in acting as a non-polar type mutation to the downstream *degU *gene. The *swrAA *gene has been reported to enhance *flgB *expression by an unknown mechanism [25]. In addition, this gene carries a frame-shift mutation in the 168 strain [26]. The transcription of the *flgB *fusion is solely driven by sigma A type RNA polymerase, as the fusion lacks a promoter for sigma D type RNA polymerase [23]. In the strain bearing functional *swrAA *at an ectopic *thrC *locus, the expression of F1D was about four-fold higher than that in the 168 (*swrAA*) strain, which is consistent with former results [25]. As unphosphorylated DegU binds to two BRs, it was expected that unphosphorylated DegU would regulate the expression of F1D. If this were the case, then only the *degU *mutation but not the *degS *mutation would have an effect on fusion expression. As shown in Figure 3A, in *swrAA *and *swrAA*^+ ^backgrounds, the *degU *mutation decreased the expression of F1D, while the *degS *mutation had little or no effect. The effect of the *degU *mutation was more prominent in the *swrAA*^+ ^background than in the *swrAA *background. These results indicated that unphosphorylated DegU positively regulates F1D fusion expression. Moreover, the *degU32 *mutation, which makes DegU-P resistant to dephosphorylation [5], reduced the expression of F1D 3-fold and 1.7-fold in the *swrAA *and *swrAA*^+ ^backgrounds, respectively. This observation is in good agreement with former results [8,9] (Figure 3B).

### DegU and SwrAA act on *flgB *expression through BR1

To determine the in vivo role of BR1 in the positive regulation of *flgB *by DegU, we constructed a fusion carrying BR1 alone (F1) and tested its expression in various genetic backgrounds (Figure 3B). As well as F1D, the expression of F1 was decreased in the *degU *but not *degS *background, suggesting positive regulation of F1 by unphosphorylated DegU. The reduced expression in the presence of the *degU *mutation and the increased expression in the presence of functional *swrAA *were abolished by introducing point mutations into the upstream and downstream half sites of the IR-like sequences in BR1 and the long spacing region (F1M1, F1M3 and F1M2, respectively) or by deleting BR1 (F2 and F3). These results demonstrated that unphosphorylated DegU binding to BR1 is required for the positive regulation of *flgB *by DegU, and for the enhancing effect of SwrAA. This result strongly suggested that at least part of the positive function of SwrAA is exercised through DegU. In fact, in the *degU *strain carrying F1D, F1DM or F1, SwrAA had little effect on the expression of *flgB*. Surprisingly, the introduction of the *degU32 *mutation into a strain carrying F1 and *swrAA *resulted in about a three-fold increase in the expression of F1, which is not consistent with the results of the expression analysis of F1D. This positive effect of *degU32 *in the *swrAA *strain was abolished by disrupting or deleting BR1, suggesting that higher cellular concentrations of DegU-P may positively regulate *flgB *expression through increased binding of DegU-P to BR1. However, this hypothesis is not in agreement with the result shown in Figure 1. We will discuss this discrepancy with respect to the binding of DegU to BR1 (Discussion).

### BR2 appears to prevent the positive regulation of *flgB *by DegU-P through BR1

To investigate the in vivo role of BR2, we constructed a F2D fusion carrying an incomplete BR1, BR2, and the core promoter, and tested its β-galactosidase activities in various genetic backgrounds. The levels of expression of F2D in all of the genetic backgrounds tested were similar to those of the F3 fusion, which should reflect strength of the core promoter of *flgB*. Moreover, the absence of effects of *degU *and functional *swrAA *in F1MD was reasonable, because F1MD is equivalent to F2D with respect to distribution of BRs. These results suggested that BR2 is not involved in positive regulation by SwrAA and unphosphorylated DegU. A comparison of the expression patterns of the F1D and F1DM fusions showed that there was essentially no difference except for the epistatic effect of the *degU32 *mutation. This suggested that BR2 could have some role to play in the negative effect of the *degU32 *mutation on F1D expression. In the presence of BR2, the *degU32 *mutation did not exhibit positive regulation of *flgB *through BR1, while, in the absence of BR2, the mutation resulted in increased fusion expression. Thus, BR2 appears to prevent the positive regulation of *flgB *by DegU-P through BR1, leading to downregulation of F1D in *degU32 *cells (Figure 3C). In addition, DegU-P bound to BR2 could not serve as a repressor, because there was no significant effect of the *degU32 *mutation on F2D expression. It should be noted that the slight reducing effect of the *degU32 *mutation on F1MD was observed in the *swrAA *background. This is due to an unknown reason.

### Transcription start site of *sacB*

To characterize the interaction of DegU-P with its targets, we selected *sacB*, which is a well-characterized gene belonging to the DegU-P regulon. The DegU-P dependency of *sacB *expression has been reported previously [27]. We determined the transcription start site of *sacB *by primer extension analysis (data not shown). A previous analysis found that the transcription start site is seven-base downstream of the newly determined site [28]. This discrepancy is due to an unknown reason. The numbering of nucleotide of *sacB *was done based on our result hereafter.

### Footprint analysis of the *sacB *promoter region

**Figure 4:**
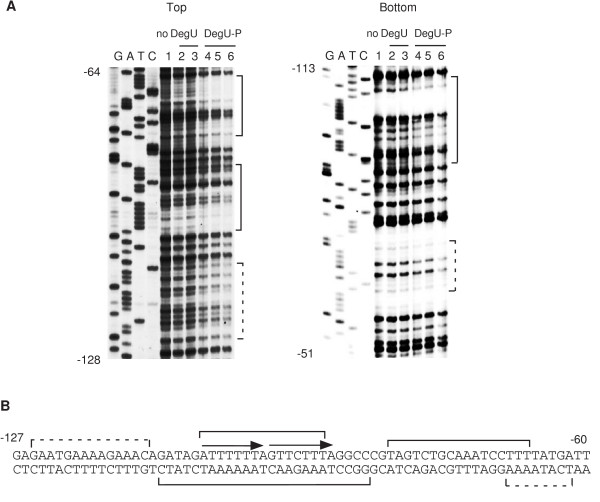
Footprint analysis of the *sacB *promoter. A. To analyze the top and bottom strands, the oligonucleotide pairs sacB-bio3 and sacB-D, and sacB-U and sacB-Pex2 were used for PCR amplification, respectively. The sequencing ladder template was generated by PCR using the oligonucleotide pair sacB-seq and sacB-U. The ladders were generated by using the primer sacB-bio3 and sacB-Pex2. The probe (40 nM) was incubated with increasing amounts of DegU and His-DegS in the presence or absence of ATP, and subjected to DNase I cleavage. The sequencing ladder is shown in lanes G, A, T and C. Lane 1. No protein; 4, 0.15 μM of DegU and 0.05 μM His-tagged DegS; 2 and 5, 0.3 μM of DegU and 0.1 μM His-tagged DegS; 3 and 6; 0.6 μM of DegU and 0.2 μM His-tagged DegS. 2 and 3, no ATP; 4, 5 and 6, 1 mM ATP. The nucleotide sequence of the promoter region is shown. The solid and dotted brackets indicate the strongly and weakly protected regions, respectively. B. Arrows above the sequences and the numbers indicate the DegU-binding motifs and the nucleotide positions relative to the transcription start site, respectively.

**Figure 5:**
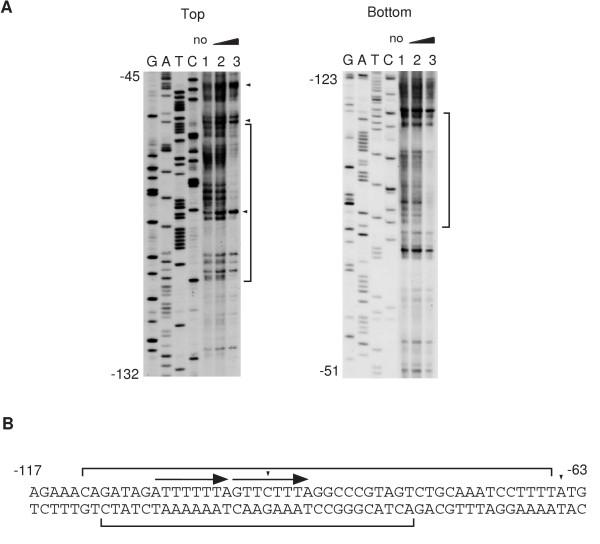
Footprint analysis of the *sacB *promoter by using His-DegU. A. Preparation of the probes and the sequencing ladders was the same as that in figure 4. The probes (40 nM) were incubated with increasing amounts of His-tagged DegU (0.3 and 0.6 μM) and subjected to DNase I cleavage. For footprint assays, we used the procedures developed for biotinylated DNA probes as described previously [47]. The sequencing ladder is shown in lanes G, A, T and C. The nucleotide sequence of the promoter region is shown. The brackets and arrowheads indicate the protected regions and hypersensitive sites, respectively. B. Arrows above the sequence indicate the DegU-binding motifs. The numbers indicate the nucleotide positions relative to the transcription start site.

To further characterize the interaction of DegU with the *sacB *promoter, we performed DNase I footprint analysis (Figure 4). Unphosphorylated DegU had no effect on DNase I cleavage of the *sacB *promoter region, while DegU-P protected the several regions on the both strands from DNase I cleavage. These results are consistent with DegU-P-dependent regulation of the *sacB *gene observed in vivo. The region containing a DR (-105 to -90) of the putative DegU-binding sequence was only protected on the both strands. In addition, we found that His-tagged unphosphorylated DegU was able to bind to the *sacB *promoter region in footprint analysis for an unknown reason (see Discussion). In the experiments using His-tagged DegU the region containing the DR was protected from DNase I cleavage (Figure 5).

### Confirmation of the *in vivo *function of the detected DR by *lacZ *fusion analysis

**Figure 6:**
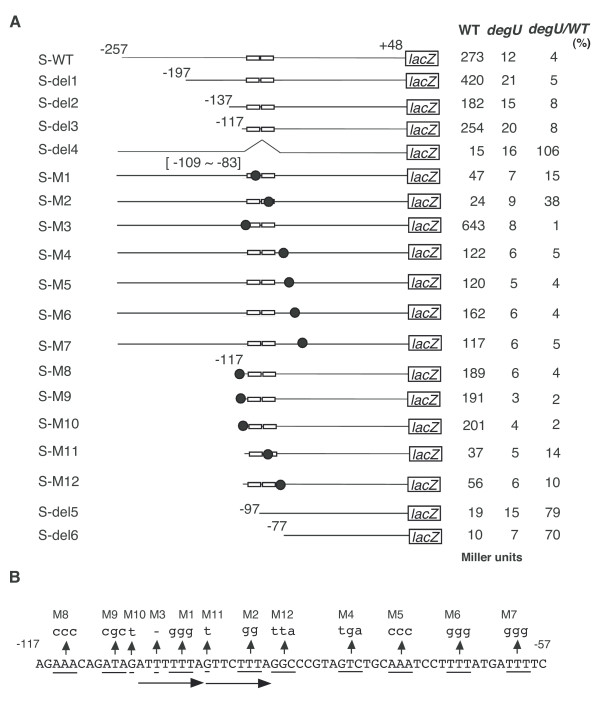
Expression of the various *sacB-lacZ *fusions. A. The 5' and 3' endpoints of *sacB-lacZ *are indicated. The open rectangles on the line indicate the DegU-binding motifs. The mutant fusions tested included the del series of fusions, in which increasingly large deletions were made from the 5' end, and the M series, in which point mutations (replaced nucleotides are shown in B. M3 is the mutation where one T residue is deleted.) were introduced (indicated by the filled circles on the line). All fusions were constructed by cloning various PCR products into pIS284. Consequently, all strains are derivatives of OAM371 (*amyE*::*sacB-lacZ*). The oligonucleotides used to construct the pIS284 derivatives are shown in Additional file 1. The β-galactosidase activities of *sacB-lacZ *were measured from cells grown in MC medium containing 1 M NaCl as described previously [45]. The activities were assayed hourly from T0 through to T4. At least three independent assays were performed and the averages of the peak values are shown. Standard deviations did not exceed 15%. The "*degU*" columns show the β-galactosidase activities of each fusion in the *degU*::Km^r ^background.

To confirm the functions of the detected DegU-binding regions *in vivo*, we performed *lacZ *fusion analysis. The expression of *sacB *is known to be regulated in two ways, namely, by DegU-P and by the antitermination system involving SacY. It has been shown previously that the upstream and downstream regions of the core promoter are required for the former and latter regulatory mechanisms, respectively, and that the deletion of the downstream region resulted in high constitutive expression of *sacB *in the absence of sucrose [27]. Thus, we only fused the upstream region of *sacB *to the *lacZ *gene. Sequential deletion of this *sacB-lacZ *fusion revealed the importance of the -117 to -97 region in the *sacB *promoter (Figure 6). We introduced various point mutations and a deletion into the -115 to -60 region, which contains the DegU-P binding sites, and examined their β-galactosidase activities. Especially, the deletion of the region containing the DR resulted in the strongest effect on *sacB *expression (S-del4) among the mutants tested. In addition, the M1 and M2 mutations disrupting the DR led to significant decreases in *sacB-lacZ *expression. These results confirmed the functionality of the DR. Furthermore the other detected DegU-binding sites did not have significant in vivo roles, because the introduced mutations to the regions showed marginal effects on transcription of the fusions. We note that the mutation located immediately downstream of the DR (S-M12) reduced fusion expression to some extent. Notably, a previous deletion study had shown that the ability of the *degU32 *mutation to enhance *sacB *expression is dependent on the -137 to -90 region of the *sacB *promoter [29]; this region contains the DR we identified. Another interesting point is that the nucleotide change that yields the S-M3 point mutant makes the sequence of the upstream motif closer to the DegU-P-binding consensus motif (Figure 6B); this led to a 2.5-fold higher level of expression of the fusion compared to that of S-WT.

### Changing the DR to an IR in the *sacB *promoter results in *degS*-independent but *degU*-dependent regulation

**Figure 7:**
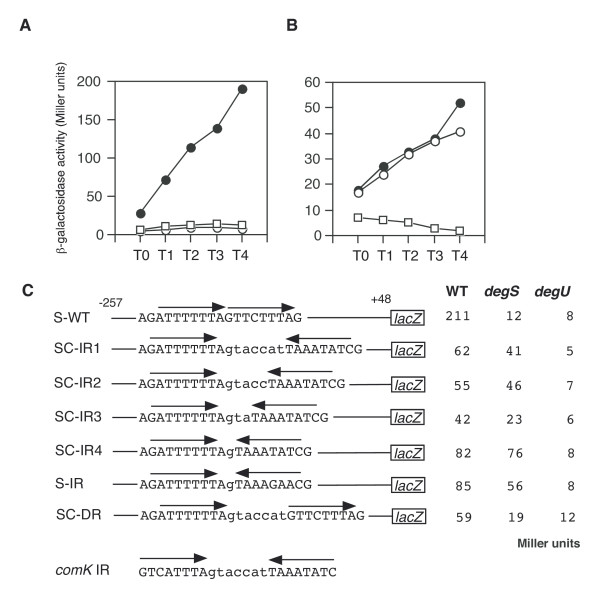
Effect of arrangement of the DegU-recognition motifs in the *sacB *promoter. All fusions constructed by cloning various PCR products into pIS284 are derivatives of the S-WT fusion and the oligonucleotides used to construct are shown in Additional file 1. The β-galactosidase activities of *sacB-lacZ *were measured from cells grown in MC medium containing 1 M NaCl as described previously [45]. A and B. Filled circles, *degS*^+ ^*degU*^+^; open circles, *degS*; squares, *degU*. The S-WT (A) and SC-IR2 (B) fusions are shown. C. The activities were assayed hourly from T0 through to T4. At least three independent assays were performed and the averages of the peak values are shown. Standard deviations did not exceed 10%. The "*degS*" and "*degU*" columns show the β-galactosidase activities of each fusion in the *degS*::Em^r ^and *degU*::Km^r ^backgrounds, respectively. Arrows above the sequences indicate the DegU-binding motifs.

**Figure 8:**
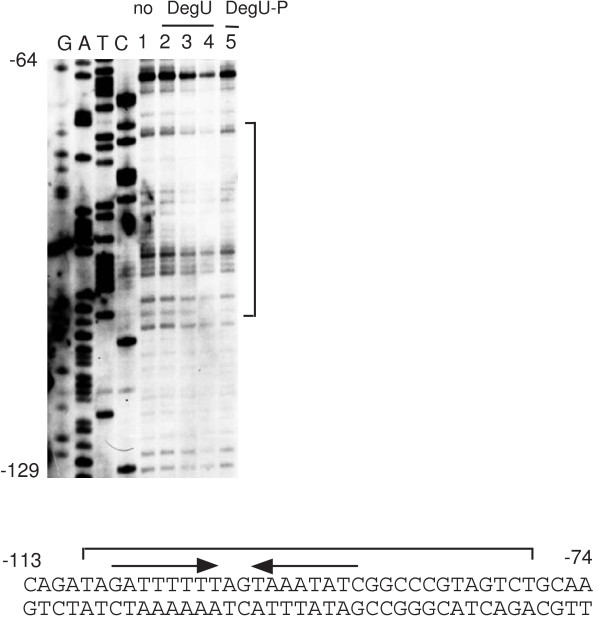
Footprint analysis of the *sacB *promoter carrying IR. To analyze the top strand, the oligonucleotide pair sacB-bio3 and sacB-D was used for PCR amplification from the plasmid carrying the SC-IR4 fusion (figure 7). The procedures for sequencing ladder generation and footprint analysis were the same as those in figure 5. The probe (40 nM) was incubated with increasing amounts of DegU and His-DegS in the presence or absence of ATP, and subjected to DNase I cleavage. The sequencing ladder is shown in lanes G, A, T and C. Lane 1. No protein; 2, 0.15 μM of DegU and 0.05 μM His-tagged DegS; 3, 0.3 μM of DegU and 0.1 μM His-tagged DegS; 4 and 5; 0.6 μM of DegU and 0.2 μM His-tagged DegS. 2, 3 and 4, no ATP; 5, 1 mM ATP. The nucleotide sequence of the promoter region is shown. The bracket indicates the protected region. Arrows above the sequences and the numbers indicate the DegU-binding motifs and the nucleotide positions relative to the transcription start site, respectively.

According to the above results, it is possible that the IR-like and the DR sequences positively regulate gene expression by unphosphorylated DegU and DegU-P, respectively. The expression of *aprE *and *sacB*, whose promoters carry a DR, is positively regulated by DegU-P (Figure 4 and Figure 6) [19]. In addition, we found recently that three DRs with zero or two-nucleotide spacing serve as positive cis-elements for DegU-P in the regulatory region of *bpr*, which is a DegU-P-regulated gene [22]. In contrast, the expression of *comK *and *flgB*, whose promoters carry IR and IR-like sequences, respectively, is regulated by unphosphorylated DegU (Figure 1 and Figure 3) [19]. Thus, we examined the effect of changing a DR to an IR on *degS*-dependent expression of the *sacB-lacZ *fusion by replacing the downstream half of the *sacB *DR by that of *comK *IR with various spacers. The expression of wild-type *sacB-lacZ *was dependent on both the *degS *and *degU *genes (Figure 7A). In the case of SC-IR2, expression of the fusion was not affected when the *degS *mutation was introduced, although the expression was still dependent on functional *degU *(Figure 7B). This strongly suggested that changing the DR to an IR results in the appearance of unphosphorylated DegU-dependent positive regulation. The other series of SC-IR fusions showed similar regulation patterns to that of SC-IR2 (Figure 7C). Next, we reversed the orientation of the downstream half site of *sacB *DR to make an artificial IR in the *sacB *promoter, which had the original sequence except for the insertion of one G nucleotide to make two-base spacing. This change rendered the expression of the SC-IR fusion resistant to the *degS *mutation to some extent. Furthermore, unphosphoylated DegU was able to bind to the IR in SC-IR4 in the footprint analysis (Figure 8). These results are further supports of our hypothesis.

Another difference between the promoters of DegU-regulated and DegU-P-regulated genes is that the IR of *comK *and IR-like sequences of *flgB *(BR1 and BR2) have long spacing regions, whereas the DRs of *aprE, bpr *and *sacB *have short spacing regions. Thus, we examined whether a long spacer between the two half sites of *sacB *DR might alter its regulation. The introduction of a long spacer corresponding to that of the *comK *IR into the DR of *sacB *(SC-DR) did not affect *degS*-dependent positive regulation of *sacB *(Figure 7C). This suggests that spacing between two half-sites would not be a critical factor for the promoter selectivity of DegU.

## Discussion

**Figure 9:**
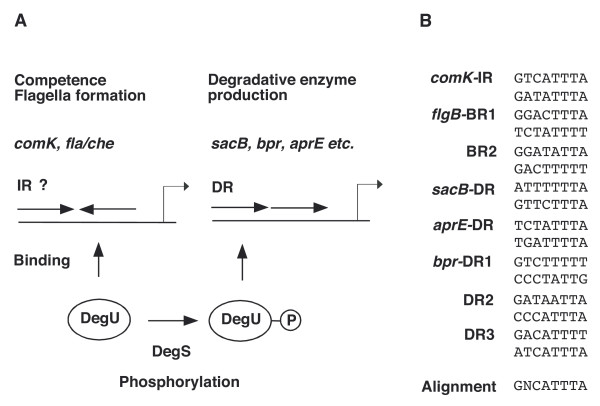
Schematic representation of the regulation of the DegU-regulon. A. Unphosphorylated DegU binds to the BRs containing the IR-like sequences in the *flgB *promoter or IR in the *comK *promoter. DegS phosphorylates DegU in response to some unknown signal. DegU-P binds to DR in the *sacB*, as well as probably to the *bpr *and *aprE *promoters. B. Alignment of DegU-recognition sequences. *comK*-IR and *aprE*-DR are shown in [19] and *bpr*-DRs are shown in [22].

This study demonstrated that while phosphorylation of DegU stimulates its binding to the DR in the *sacB *promoter, it inhibits DegU-binding to the artificial IR of the same promoter. These results strongly suggested that DegU-P prefers binding to DR but not to IR (Figure 9A). This hypothesis is consistent with the observation that phosphorylation of DegU abolished its binding to BR1 containing the IR-like sequence in the *flgB *promoter under the condition used. DegU-P, however, could bind to the BR2 in *flgB *and the *degU32 *mutation resulted in positive regulation of the F1 fusion through BR1, suggesting that DegU-P could bind to this region when cellular concentrations of DegU-P were high enough. We note that this positive regulation of the *degU32 *mutation was observed only in the *swrAA *background. The possible binding of DegU-P to the *flgB *promoter may be an exception to the hypothesis and remains to be elucidated.

Notably, DegU belongs to the NarL response regulator family, which is characterized by a classical helix-turn-helix domain that recognizes IRs of the same motifs [30]. However, NarL has also been reported to bind to differently arranged motifs, namely, DRs [31]. Indeed, another report has shown that the NarL-binding sites in various promoters are differently arranged as direct repeats, monomers, or inverted or divergent repeat [32]. It is quite possible that the binding to differently arranged motifs requires different protein-protein interactions within the putative dimers of DegU, which might be regulated by phosphorylation. Global analysis of the DegU regulon has identified many genes other than the genes analyzed in this study [8,9]. It would be of interest to determine how the DegU motif is arranged in the promoters of these genes as this may further enhance our understanding of DegU regulation.

On the basis of the DegU-binding sites we have identified, we propose that 5'-GNCATTTA-3' is the consensus DNA-binding sequence of DegU and DegU-P (Figure 9B). Supporting this is a previous study, which also proposed a putative DegU consensus sequence on the basis of genetic analysis of the *wapA *promoter [7]: our newly proposed consensus is consistent with the important sequences for DegU-binding to the *wapA *promoter. The DegU-binding sequences that we identified are rather degenerate, and we speculate that this may be a common feature of the sequences recognized by other global regulators. Therefore, it may be difficult to identify DegU-binding sequences by inspecting of the upstream regions of DegU-regulon genes. For example, there is DegU-consensus-like sequences in the *sacB *promoter region (-72 to -57), and the region appeared to be protected from DNase I cleavage to some extent. The mutational analysis, however, revealed that the sequences do not serve as a functional cis-element for DegU-P (S-M6 and S-M7 in Figure 6). Unknown features other than the primary sequence of the DNA target may be involved in DegU-P regulation.

We observed that His-tagged unphosphorylated DegU bound to the promoter regions of *aprE *and *sacB *(Figure 5) [14,19]. This might mean that His-tagged unphosphorylated DegU can bind to DR. This characteristic is different from that observed for intact unphosphorylated DegU. It was reported that His-tagged Spo0A was phosphorylated in *E. coli*, resulting in an isolation of Spo0A-P without any phosphorylation reaction [33]. Thus, it is possible that His-tagged DegU purified from *E. coli *might undergo phosphorylation and serve as DegU-P. The possibility is unlikely, however, because a half-life of DegU-P has been reported to be less than 90 min due to an intrinsic phosphatase activity of DegU [5]. It has been shown that the addition of the His-tag to the C-terminus of *Salmonella *PhoP response regulator affects its biochemical properties and conformation with respect to its dimer formation and DNA-binding ability [34]. Addition of His-tag at the N-terminus of DegU might alter its conformation as in the case of *Salmonella *PhoP.

In our analysis of the mechanisms regulating *flgB *expression, we observed that SwrAA requires DegU-binding to BR1 to enhance *flgB *expression. While the exact function of SwrAA is not yet understood, it is known that *swrAA *is required for swarming motility [26,35], γ-poly-glutamic acid synthesis [36] and enhancing of the transcription of the large *fla/che *operon in a non-laboratory strain [25]. The result that SwrAA stimulates the *fla/*che transcription in a DegU-dependent manner led us to a speculation that SwrAA might facilitate DegU-binding to its target promoter or modulate the DegU function. We noted that if SwrAA functions to enhance the expression of some DegU-dependent genes, it remains to be determined how SwrAA focuses specifically on its targets among the many genes that are regulated by DegU.

Amati *et al*. showed that the downstream region from the *flgB *transcription start site containing BR2 did not interact with DegU [24]. This result is inconsistent with our footprint data (Figure 1 and Figure 2). We do not know the reason for this discrepancy. In addition, they claimed that DegU-binding to the upstream region resulted in repression of *ylxF*, which is the ninth gene of the *fla/che *operon. Our result clearly showed that the expression of the F1 fusion containing only BR1 was increased in *degU32 *cells (Figure 3B). The different interpretation between their results and ours might be due to the use of *ylxF-lacZ *in their analysis. DegU-P bound to BR1 could be somehow dysfunctional in the presence of BR2. The regulatory mechanism involving the two BRs remains to be solved, although we speculate that DegU-P bound at each region might interact together through DNA-looping, leading to the abolishment of the positive effect of DegU-P through BR1. As a result, in the regulatory region carrying both regions, the *degU32 *mutation results in a decrease in *flgB *expression. This could be caused by a decrease in the cellular concentrations of unphosphorylated DegU, but not in the repressor function of DegU-P at BR2, as BR2 did not serve as a functional cis-acting site in the absence of BR1. DegU-P at BR2 might rather work as an anti-activator.

We found that unphosphorylated DegU is a positive regulatory factor of the *flgB *promoter, which is the first gene in the *fla/che *operon (including the *sigD *gene that encodes sigma D factor). It was reported that disruption of *degU *decreases the expression of *sigD *[37], which is governed by the upstream *flgB *promoter [23,38,39]. Our findings are consistent with this observation.

Recently it has been reported that swarming motility and the *flgB *expression in the non-laboratory strain requires DegS-independent low-level of DegU-P [40,41]. These observations may be consistent with our findings, since unphosphorylated DegU (or DegU phosphorylated at a low level) may positively regulate the expression of the *fla/che *operon. To further characterize the DegS-independent phosphorylation of DegU, a study using a DegU mutant lacking the phosphorylation site should be needed.

## Conclusion

This study demonstrated that DegU positively regulates *flgB *and *sacB *via the BRs containing the IR-like sequences and the DR present in their promoter regions, respectively (Figure 9). In, addition, we showed that DegU-P prefers binding to DR but not to IR in the regulatory region of the *sacB *promoter.

## Methods

### Bacterial strains and culture media

All the *B. subtilis *strains used for this study are listed in Table 1. One-step competence medium (MC) was used [6]. For DNA manipulation, *Escherichia coli *cells were grown in LB medium. The concentrations of antibiotics used have been described previously [42].

**Table 1:** Strains and plasmids used for this study.

Strain	Genotype	Reference or source
168	*trpC2*	Laboratory stock
IFO3335	Non-laboratory strain	Laboratory stock
IA95	*trpC2 degU32*	BGSC
OAM392	*trpC2 thrC *::*swrAA *N (Sp^r^)	This study
TT714	*trpC2 leuC7 degU *(Km^r^)	[46]
TT719	*trpC2 leuC7 ΔdegS *(in frame deletion)	[46]
OAM369	*trpC2 ΔdegS *(in frame deletion, *yvyE *::Cm^r^::Em^r^)	This study
OAM385	*trpC2 amyE *::*flgB-lacZ *1D (Cm^r^)	This study
OAM386	*trpC2 amyE *::*flgB-lacZ *1D (Cm^r^) *thrC *::*swrAA *N (Sp^r^)	This study
OAM387	*trpC2 amyE *::*flgB-lacZ *1D (Cm^r^) *degU *(Km^r^)	This study
OAM388	*trpC2 amyE *::*flgB-lacZ *1D (Cm^r^) Δ*degS *(Em^r^)	This study
OAM389	*trpC2 amyE *::*flgB-lacZ *1D (Cm^r^) *thrC *::*swrAA *N (Sp^r^) *degU *(Km^r^)	This study
OAM390	*trpC2 amyE *::*flgB-lacZ *1D (Cm^r^) *thrC *::*swrAA *N (Sp^r^) Δ*degS *(Em^r^)	This study
OAM391	*trpC2 amyE *::*flgB-lacZ *1D (Cm^r^) *degU32*	This study
OAM371	*trpC2 amyE *::*sacB-lacZ *S-WT (Cm^r^)	This study
OAM372	*trpC2 amyE *::*sacB-lacZ *S-WT (Cm^r^) *degU *(Km^r^)	This study
OAM373	*trpC2 amyE *::*sacB-lacZ *S-WT (Cm^r^) Δ*degS *(Em^r^)	This study
OAM393	*trpC2 amyE *::*sacB-lacZ *SC-IR2 (Cm^r^)	This study
OAM394	*trpC2 amyE *::*sacB-lacZ *SC-IR2 (Cm^r^) *degU *(Km^r^)	This study
OAM395	*trpC2 amyE *::*sacB-lacZ *SC-IR2 (Cm^r^) Δ*degS *(Em^r^)	This study

Plasmid	Description	Reference or source

pDG-swrAAN	pDG1731 carrying *swrAA *N	This study
pCa-yvyE	pCA191 carrying a part of *yvyE *and a promoter of *degSU*	K. Kobayashi
pCm::Em^r^	Ampicillin and erythromycin resistance	[48]
pTYB-degU	pTYB2 carrying *degU*, ampicillin resistance	This study
pET-degS	pET28b carrying *degS*, kanamycin resistance	K. Kobayashi
pDG-His-degU	Plasmid carrying T5 promoter-His6-*degU*, ampicillin resistance	[19]
pIS284	Insertion vector to *amyE*, chloramphenicol resistance, *lacZ*	I. Smith
pIS284-flgB	pIS284 carrying a promoter region of *flgB*	This study
pIS284-sacB	pIS284 carrying a promoter region of *sacB*	This study

### Plasmid construction

Synthetic oligonucleotides were commercially prepared by the Tsukuba Oligo Service (Ibaraki, Japan). The plasmids and oligonucleotides used in this study are listed in Table 1 and Additional file 1, respectively. We used total DNA from *B. subtilis *168 as the PCR template unless otherwise indicated. To construct pDG-swrAN, a PCR product produced by using swrA-FA and swrA-FB and total DNA from *B. subtilis *IFO3335 was digested by *Bam*HI and *Eco*RI and cloned into PDG1731 treated with the same restriction enzymes [43]. To construct pTYB-degU, a PCR product produced by degU-Chi-N and degU-Chi-S was digested by *Nde*I and *Sma*I and cloned into pTYB2 treated with the same restriction enzymes. To construct pCa-yvyE, a PCR product produced by using yvyE-F1 and yvyE-R1 was digested by *Hin*dIII and *Bam*HI and cloned into pCA191 treated with the same restriction enzymes [44]. To construct pIS284-flgB and pIS284-sacB, PCR products were prepared by using the primer pairs, flgB-F2 and flgB-DD, and sacB-U and sacB-D, respectively. The DNA fragments were digested with *Bam*HI and *Hin*dIII and ligated into the similarly-digested pIS284 plasmid. The resultant plasmids were transformed into 168 after linearization by *Pst*I digestion, thereby generating the strain carrying F1D and S-WT, respectively. The del series of *sacB-lacZ *fusions, the F1 to F3 derivatives, and F2D were constructed by generating PCR products using oligonucleotides corresponding to different 5'-termini or 3' termini of the fusions and cloning these fragments into pIS284. The M series of *flgB-lacZ *and *sacB-lacZ *fusions and the SC series of *sacB-lacZ *fusions as well as S-IR were constructed by cloning mutagenized PCR products into pIS284. Site-directed mutagenesis was performed by an oligonucleotide-based PCR method as described previously [45]. S-del4 was constructed by site-directed mutagenesis using oligonucleotides sacB-del-1, sacB-del-8, sacB-U and sacB-D. S-M3 was obtained by PCR error during the construction of S-WT. The resulting fusion-bearing plasmids were used to transform 168. The sequences cloned into all plasmids were confirmed.

### Purification of soluble His-tagged DegS, His-tagged DegU and intact DegU proteins

The recombinant His-tagged DegS, His-tagged DegU and chitin-binding domain (CBD)- and intein-fused DegU proteins were induced in *E. coli *BL21 carrying pET-degS, M15 carrying pRep4 and pDG-His-degU, and BL21 carrying pTYB-degU by previously described methods [14]. His-tagged DegS was produced as insoluble protein and its pellets were resuspended in 2.5 ml of buffer A [6 M guanidine-HCl, 150 mM NaCl, and 50 mM Tris-HCl (pH 7.6)] and left for 1 hr at room temperature. Two ml of 50% Ni-NTA resin (Qiagen) equilibrated by buffer A were added to the sample, which was then shaken gently for 45 min at room temperature. The resultant suspension (about 5 ml) was packed in a mini-column, washed first with 10 μl of buffer A and then with 5 ml of buffer B [150 mM NaCl and 50 mM Tris-HCl (pH 7.6)] containing 8 M urea. Renaturation of His-tagged DegS was performed by removing urea, which was done in a step-wise fashion by passing 5 ml each of buffer B containing 6 M, 4 M and 2 M urea through the column. Finally, the column was washed with 5 ml of buffer B containing 10 mM imidazole. The protein was then eluted with buffer B containing 0.5 M imidazole. His-tagged DegU was produced as a soluble protein in *E. coli *and purification was performed by step-wise elution from a Ni-affinity column with imidazole as described previously [14]. CBD- and intein-fused DegU was purified by using chitin-coupled resin and autoactivating intein by DTT according to the manufacturer's recommendations (New England Biolab inc). After SDS-PAGE analysis of the fractions, the purified proteins were dialyzed against TEDG buffer [46]. Aliquots of the purified proteins were stored at -80°C.

### Footprint assays using DegU-P

Phosphorylation of DegU and the binding of DegU-P to the probe DNA were carried out by incubating intact DegU and His-tagged DegS at 25°C for 30 min (molar ratio 3:1) with a biotinylated DNA probe, i.e., in a buffer containing protein solution (TEDG buffer). The final concentration of the reaction mixture (65 μl) for the footprint analysis was as follows; 10 mM (NH4)2SO4, 1 mM DTT, 0.2% Tween20, 5 mM MgCl2, 31 mM Tris-HCl (pH 7.5), 0.3 mM EDTA, 44 mM KCl, 1 μg poly dI-dC and 1 mM ATP. Immediately at the end of the reaction, 4 units of DNase I (Roche, Indianapolis, USA) in 7. 5 μl buffer containing 6% glycerol, 10 mM Tris-HCl (pH 7.5), 50 mM NaCl, 5 mM MgCl2, 1 mM DTT and 1 mg bovine serum albumin was added. The reaction mixture was left at room temperature for 5 min and then subjected to phenol extraction and subsequent ethanol precipitation after the addition of stop solution (0.1% SDS, 20 mM EDTA, 200 mM NaCl, 40 μg/ml tRNA). After the addition of a loading dye, the samples were applied onto a 6% polyacrylamide gel. The probes were prepared by PCR using the oligonucleotide pairs indicated in Additional file 1. Detection of biotinylated DNA and preparation of sequencing ladder were done by using the methods described previously [14].

## Authors' contributions

KT carried out protein purification and footprint analysis. MO carried out construction of plasmids and strains, and in vivo *lacZ *analysis and wrote the paper. All the authors have read and approved the final manuscript.

## Supplementary Material

Additional file 1 Oligonucleotides used for this study. Oligonucleotide sequences used for this study. Click here for file
